# Identification, cloning and characterization of a novel 47 kDa murine PKA C subunit homologous to human and bovine Cβ2

**DOI:** 10.1186/1471-2091-7-20

**Published:** 2006-08-04

**Authors:** Ane Funderud, Heidi H Henanger, Tilahun T Hafte, Paul S Amieux, Sigurd Ørstavik, Bjørn S Skålhegg

**Affiliations:** 1 Department of Nutrition Research, Institute of Basic Medical Sciences, University of Oslo, PO Box 1046 Blindern, 0317 Oslo, Norway; 2 Department of Pharmacology, University of Washington School of Medicine, PO Box 357750, Seattle, WA 98195-7750, USA

## Abstract

**Background:**

Two main genes encoding the catalytic subunits Cα and Cβ of cyclic AMP dependent protein kinase (PKA) have been identified in all vertebrates examined. The murine, bovine and human Cβ genes encode several splice variants, including the splice variant Cβ2. In mouse Cβ2 has a relative molecular mass of 38 kDa and is only expressed in the brain. In human and bovine Cβ2 has a relative molecular mass of 47 kDa and is mainly expressed in lymphoid tissues.

**Results:**

We identified a novel 47 kDa splice variant encoded by the mouse Cβ gene that is highly expressed in lymphoid cells. Cloning, expression, and production of a sequence-specific antiserum and characterization of PKA catalytic subunit activities demonstrated the 47 kDa protein to be a catalytically active murine homologue of human and bovine Cβ2. Based on the present results and the existence of a human brain-specifically expressed Cβ splice variant designated Cβ4 that is identical to the former mouse Cβ2 splice variant, the mouse splice variant has now been renamed mouse Cβ4.

**Conclusion:**

Murine lymphoid tissues express a protein that is a homologue of human and bovine Cβ2. The murine Cβ gene encodes the splice variants Cβ1, Cβ2, Cβ3 and Cβ4, as is the case with the human Cβ gene.

## Background

Cyclic AMP dependent protein kinase (PKA) is a holoenzyme that consists of a regulatory (R) subunit dimer and two catalytic (C) subunits [1]. The enzyme is activated when two cAMP molecules bind each R subunit, causing a conformational change that releases the C subunits [2]. Different isoforms of both R and C subunits exist. Four separate R subunit genes designated RIα, RIβ, RIIα and RIIβ have been identified and characterized in all primates investigated [3,4]. In most species investigated three genes encoding the C isoforms have been identified and designated Cα, Cβ and PrKX. PrKX is an X chromosome encoded protein kinase [5] that has, despite low homology to Cα and Cβ, been identified as a PKA C subunit with the ability to release the R subunit in a cAMP-dependent fashion [1]. A murine homologue of PrKX termed PKARE has also been described [6]. Moreover, in human, an intron-less gene transcribing the mRNA for Cγ has been identified. Cγ represents a testis-specific retroposon [7]. In the mouse a C subunit pseudogene has been identified and designated Cx [8].

Several splice variants of Cα and Cβ, arising from alternative splicing of the first exon in the Cα and Cβ genes, are responsible for further diversity. The Cα gene encodes two splice variants (Cα1 and Cαs/Cα2) in mouse [9], human [10] and sheep [11]). In human 10 different splice variants encoded by the Cβ gene have been identified and called Cβ1, Cβ2, Cβ3, Cβ4, Cβ4ab, Cβ4abc, Cβ3ab, Cβ3abc, Cβ3b and Cβ4b [12]. In the mouse and bovine three and two Cβ splice variants have been identified, respectively. In bovine they are designated Cβ1 and Cβ2 [13,14] and in mouse they are designated Cβ1, Cβ2 and Cβ3 [15,16]. Cα1 and Cβ1 are ubiquitously expressed, while the other splice variants show a more tissue restricted expression pattern. CαS/Cα2 is solely expressed in sperm cells [10,11]. Whereas expression of the human Cβ2 splice variant is enriched in lymphoid tissues [17,18], it shows a more widespread pattern of expression in the bovine [14] and is restricted to the brain in mice [16]. The majority of the other Cβ splice variants identified, which include human and murine Cβ3 and human Cβ4 as well as the human Cβ3 and Cβ4 abc variants, are only detected in nerve cells and the brain [12,16,17].

A number of reports have demonstrated that both the Cα1 and Cβ1 isoforms have a relative molecular mass of approximately 40 kDa when analysed by SDS-PAGE [15]. In contrast, human and bovine Cβ2 have a relative molecular mass of 47 kDa [14,18] whereas in the mouse it is 38 kDa [16]. Both human and murine Cβ3 are 38 kDa, as is also the case with human Cβ4 [12,16,17]. For the human Cβ3 and Cβ4 abc variants, the relative molecular masses have not been determined.

We investigated the expression of C subunits in various murine tissues and identified two immunoreactive proteins of approximately 40 and 47 kDa. Whereas the 40 kDa band was ubiquitously expressed, the 47 kDa band was restricted to lymphoid tissues such as thymus, spleen and lymph node. Based on this we examined the activity, level and apparent molecular sizes of Cβ splice variants in immune cells. Using spleen cells from wild type and mice mutant for the Cβ gene we demonstrated that the 40 kDa band was Cβ1 whereas the 47 kDa protein was the murine homologue of human and bovine Cβ2. The novel murine Cβ2 was shown to be an active protein kinase that was immunodetected by an antiserum made against the human Cβ2 specific sequence. Based on this, together with sequence homology searches and sequence comparisons, we conclude that the previously reported murine Cβ2 is a homologue of human Cβ4. Thus, the murine Cβ gene encodes the splice variants Cβ1, Cβ2, Cβ3 and Cβ4, as is the case with the human Cβ gene.

## Results

### Murine spleen cells express Cα1, Cβ1 and a novel 47 kDa Cβ splice variant enriched in lymphoid tissues

**Figure 1:**
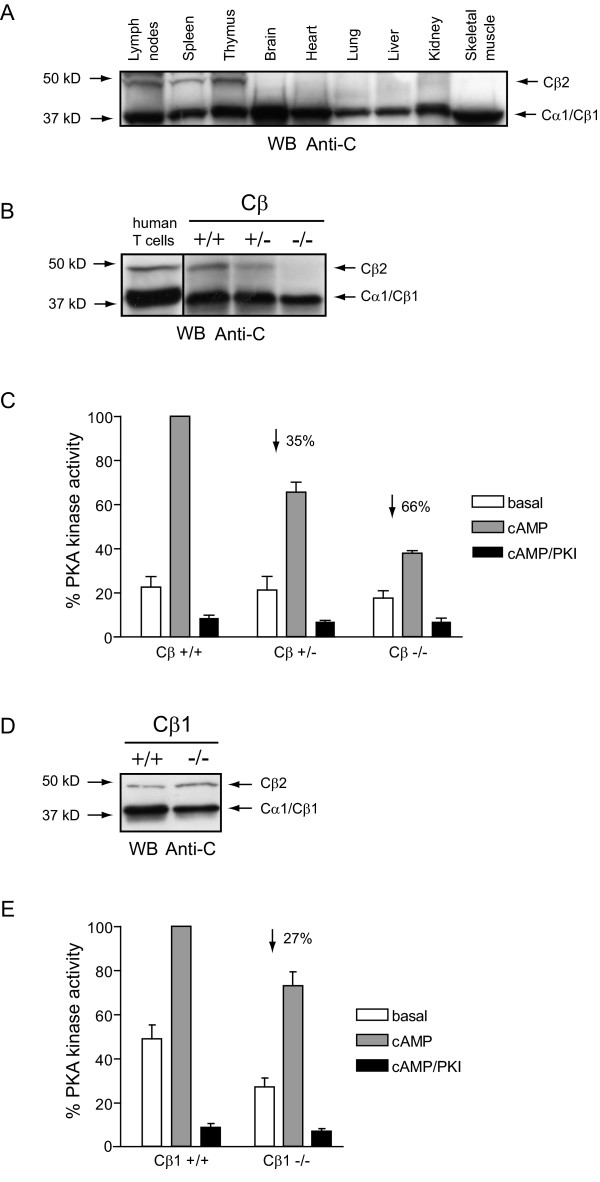
**C subunit identity and PKA specific kinase activity in murine spleen cells**. **(A) **SDS-PAGE (12.5 % gels) and anti-C immunoblotting of lysates (90 μg/lane) from lymph nodes, spleen, thymus, brain, heart, lung, liver, kidney and skeletal muscle from a WT mice. Arrows indicate molecular mass standards (left) and C subunit identities (right). **(B) **SDS-PAGE (12.5 % gels) and anti-C immunoblotting of cell lysates (60 μg/lane) from human T cells and from wild type (Cβ+/+), Cβ heterozygote (Cβ+/-) and Cβ knockout (Cβ-/-) mice. Arrows indicate molecular mass standards (left) and C subunit identities (right). **(C) **PKA-specific phosphotransferase activity in spleen lysates (1 μg/ml) isolated from wild type (Cβ+/+), Cβ heterozygote (Cβ+/-) and Cβ knockout (Cβ-/-) mice. Activity was measured in the absence (empty bars, basal) or presence (gray bars, cAMP) of 5μM cAMP or both cAMP and PKI (black bars, cAMP/PKI). Activities in the extracts from the mutant mice are given relative to the activity in the lysates from wild type cell lysate, which are set to 100 %. Bars represent mean activities of three experiments ± standard deviation. Percentage reductions in activities were calculated after subtraction of non specific kinase activity (black bars) and are indicated with an arrow. **(D) **SDS-PAGE (12.5 % gels) and anti-C immunoblotting of spleen lysates (60 μg/lane) from wild type (Cβ1+/+) and Cβ1 knockout (Cβ1-/-) mice. Arrows indicate molecular mass standards (left) and C subunit identities (right). **(E) **PKA-specific phosphotransferase activity in spleen lysates (1 μg/ml) from wild type (Cβ1+/+) and Cβ1 knockout (Cβ1-/-) mice were measured and presented as described in C. Bars represent mean activities of three experiments ± standard deviation.

When lysates made from various murine tissues were separated by SDS-PAGE and immunostained with a commercially available pan C antibody (Figure 1A), a 40 kDa band was detected in all tissues. In addition to this ubiquitously expressed band a weaker band of 47 kDa was detected, that seemed to be restricted to lymphoid tissues, as it could only be detected in lymph nodes, spleen and thymus. No expression was detected in brain, heart, lung, liver, kidney and skeletal muscle.

To investigate identity of these C immunoreactive bands, spleen cell lysates from wild type (WT) mice and mice that were heterozygous (+/-) or homozygous (-/-) for a null mutation in the Cβ gene [19] and a human T cell lysate were immunoblotted with the pan C antibody (Figure 1B). In the human T cell lysate two immunoreactive bands of 40 and 47 kDa were identified. These protein bands have previously been shown to be Cα1 (40 kDa) and Cβ2 (47 kDa) [18]. The 47 kDa immunoreactive protein band was detected in the spleen cell lysate from WT and Cβ+/- mice, but was absent in the Cβ-/- lysate, implying that it represents a Cβ splice variant. Furthermore, in the Cβ-/- lysate, the 40 kDa protein band appeared weaker in intensity. This was confirmed by densitometric scanning of the 40 kDa bands in Figure 1B (results not shown) which revealed a 40 % reduction in intensity of the 40 kDa band in the Cβ-/- lysate. Together this implies that Cβ1 (40 kDa) may be expressed in mouse spleen cells.

Spleen cell lysates from WT and Cβ mutant mice were also tested for PKA-specific phosphotransferase activity by incorporation of ^32^P from [γ-^32^P]-ATP into the PKA-specific substrate Kemptide (Figure 1C). Phosphotransferase activity was also measured in the presence of PKI to demonstrate that kinase activity was PKA-specific (black bars, cAMP/PKI). PKA-specific activity was reduced by 35 and 66 % in Cβ+/- and Cβ-/- mice, respectively, compared to WT (Cβ+/+) mice. This indicates that Cβ contributes significantly to PKA activity in mouse spleen cells.

To further investigate the identity of the 40 and 47 kDa protein bands, we analysed C subunit expression and PKA kinase activity in mice lacking the Cβ1 splice variant (Cβ1-/-) [20]. Figure 1D demonstrates that the 47 kDa C subunit immunoreactive band is present in cell lysates from both WT and Cβ1-/- mice. It should however be noted that the 40 kDa protein band was clearly weaker in intensity in lysate from Cβ1-/- mice. Densitometric scanning indicated that this reduction was of similar magnitude (approximately 30 %, results not shown) as that monitored in the Cβ-/- mice. Together with the fact that we also measured a 27 % reduction in PKA kinase activity in the Cβ1-/- mice lysates (Figure 1E) this demonstrated that Cβ1 is expressed in mouse spleen cells. Finally, the fact that reduction in PKA kinase activity in the Cβ-/- was approximately twice as much at that observed in the Cβ1-/- (66 % as opposed to 27 %) strongly implies that the lymphoid tissue specific 47 kDa protein is a Cβ splice variant and that it contributes significantly to PKA kinase activity in mouse spleen cells.

### Cloning and expression of a novel catalytically active murine Cβ splice variant homologous to human Cβ2

**Figure 2:**
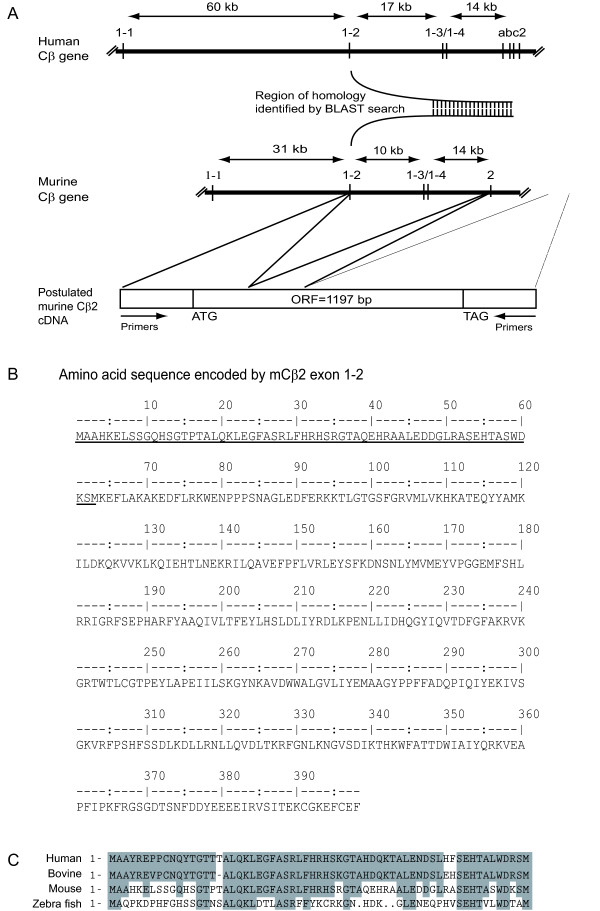
**Identification of a novel mouse cDNA sequence encoding a Cβ splice variant homologous to human Cβ2**. **(A) **Schematic representation of the human (upper) and murine (lower) Cβ gene. The intron sizes between exons (vertical lines) are indicated by horizontal arrows. A region of high similarity (identified by BLAST (tblastn) homology search in the published mouse genome) to human exon 1–2 is indicated between the genes (bent/vertical lines). A hypothetical cDNA sequence is indicated, with an 1197 base pair open reading frame (ORF 1197 bp), and a start (ATG) and stop (TAG) translation codon for murine Cβ2 is given, based on the identified sequence and the known mouse Cβ cDNA sequence (bottom). **(B) **Deduced amino acid sequence of mouse Cβ2 shown in one letter code. The Cβ2 splice variant specific sequence of 62 amino acids is underscored. **(C) **Cβ2 specific sequences from human, bovine, mouse and zebrafish were aligned and amino acid sequences conserved between species were boxed. Compared to the level of conservation in the catalytic core of C subunits, the Cβ2 specific sequence shows a lesser degree of conservation.

Our observations that murine spleen cells express a 47 kDa anti-C immunoreactive protein that is absent in the Cβ-/- animals, and co-migrates with human Cβ2, suggest that the mouse expresses a Cβ2 subunit homologous to the human Cβ2 subunit. In order to investigate this, we initially performed a homology search in the National Center for Biotechnology Information (NCBI) BLAST (tblastn) search tool using the human Cβ2 specific peptide sequence. We identified a region with high sequence similarity to the human exon 1–2 sequence in the published mouse genome [21]. This sequence was located in the mouse Cβ gene upstream of exon 2 (Figure 2A). Based on this information and the fact that all C subunits identified so far only differ in the domain encoded by alternative use of exon 1, we postulated a hypothetical cDNA sequence using this mouse exon 1–2 sequence and the known murine Cβ cDNA sequence [15] (Figure 2A, bottom). To confirm mRNA expression of this sequence, oligonucleotide primers corresponding to the 5'-untranslated and 3'-untranslated regions of the cDNA were synthesised and used for reverse transcriptase PCR of total RNA from mouse thymus since Cβ2 expression has earlier been reported to be enriched in human thymus [17]. PCR products were subcloned into pCR-Blunt-II-TOPO vector. Nucleotide sequencing (Medigenomix, Germany) and amino acid sequence deduction (Figure 2B), followed by comparison of the unique N-terminal end [17,22] with corresponding sequences in human, bovine, mouse and zebra fish (Figure 2C), confirmed the novel sequence as the murine Cβ2 homologue.

**Figure 3:**
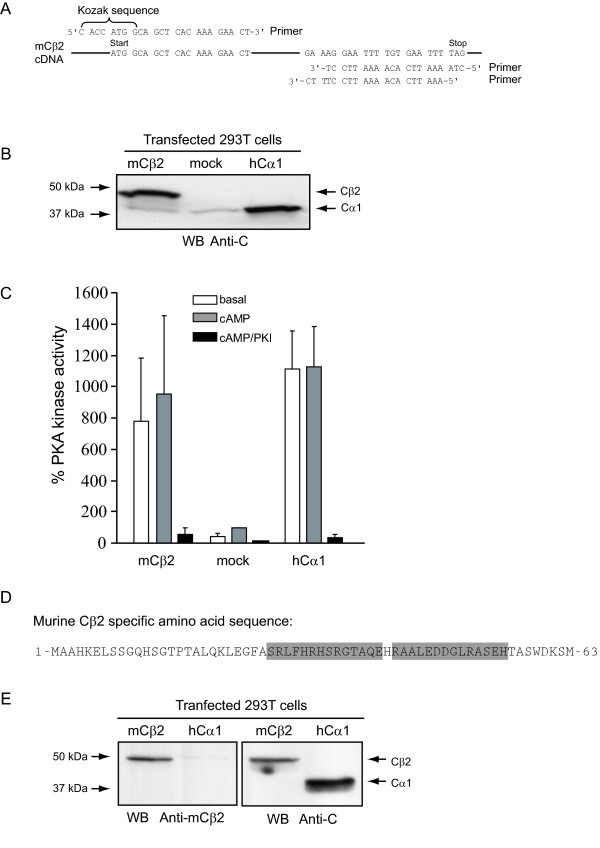
**Murine Cβ2 cDNA encodes an active protein kinase of 47 kDa recognized by a Cβ2-specific antiserum**. **(A) **Primers used to amplify mouse Cβ2 cDNA for insertion into pENTR/D-TOPO (Gateway system, Invitrogen). The upper 5'-end primer (upper sequence left) was designed to produce an insert with a Kozak sequence associated with the start codon (ATG). Two different 3'-end primers (lower right two sequences) were designed in order to include or exclude the stop codon (TAG). In the absence of the stop codon, the mouse Cβ2 would be expressed with a C-terminal tag, which is contained in the vector (Gateway, Invitrogen). Mouse Cβ2 cDNA was PCR amplified and subcloned into the pcDNA-DEST40 expression vector to yield pcDNA-DEST40 mCβ2. **(B) **HEK 293T cells (3.5 × 10^5 ^cells/ml) were transfected with 2μg DNA/ml of pcDNA-DEST40mCβ2 (mCβ2), mock-transfected (mock), or transfected with pEF-DEST51hCα1 (hCα1) for 24 h. Cells were lysed and analysed by SDS-PAGE and anti-pan C immunoblotting. Note that mCβ2, but not mock or hCα1 transfected cell extracts, contained an anti-C immunoreactive protein of 47 kDa. All lanes revealed a 40 kDa protein band which was most intense in cells transfected with pEF-DEST51hCα1 **. (C) **The same lysates were analysed for PKA-specific kinase activity in the absence (empty bars, basal) or presence (gray bars, cAMP) of 5μM cAMP, or both cAMP and PKI (black bars, cAMP/PKI). Activities were calculated relative to the activity of the mock-transfected lysate, which was set to 100 %. Bars represent mean activities of three experiments ± standard deviation. **(D) **The sequence of the two mCβ2 specific peptides (boxed) used to immunize rabbits to make mCβ2 specific antiserum. **(E) **Two rabbits were co-immunized with the two peptides and the resulting immune sera tested for immunoreactivity and specificity using cell extracts of HEK 293T cell transfected with mCβ2 (pcDNA-DEST40mCβ2) or hCα1 (pEF-DEST51hCα1). Left panel: The mCβ2 serum recognized a 47 kDa immunoreactive protein in mCβ2 but not in hCα1 transfected cell lysates. No cross-reactivity to human Cα1 could be observed. Right panel: Immunoblotting of the same lysates with a pan anti-C antibody revealed a 47 and a 40 kDa immunoreactive protein in the two extracts respectively, confirming expression of transfected constructs.

It has previously been demonstrated that a cDNA encoding the bovine Cβ2 was not active when transfected into CHO cells [22]. To determine if the mouse Cβ2 cDNA encodes an active protein kinase, the cDNA was amplified by PCR and subcloned into pcDNA-DEST40 (Gateway Technology System, Invitrogen). To optimize for efficient protein expression, a Kozak sequence was introduced in the 5'-end. Moreover, two different 3'-ends were made, one including and one excluding a stop codon to facilitate native and tagged expression, respectively (Figure 3A). The new plasmid was designated pcDNA-DEST40mCβ2 and transfected into HEK 293T cells. As a positive control pEF-DEST51Cα1, which expresses human Cα1 (hCα1), was used. Mock transfected cells served as a negative control. Twenty-four hours after transfection cells were lysed and subjected to immunoblotting using a pan C antibody. This revealed expression of an immunoreactive protein of 47 kDa in cells transfected with mCβ2, and a 40 kDa protein in cells transfected with hCα1. A weak 40 kDa band could also be seen in mCβ2 and mock transfected cell lysates (Figure 3B), representing endogenous Cα1. In order to test for enzyme activity of the expressed Cβ2, the same cell lysates were analysed for PKA-specific phosphotransferase activity against Kemptide. This demonstrated a more than 10-fold increased enzyme activity in the cells transfected with mCβ2 and hCα1 compared to the mock-transfected cells (Figure 3C). The PKA specific inhibitor PKI abolished this activity, implying that the activity observed is PKA-specific and that mouse Cβ2 is an active PKA enzyme. It should be noted that the C-terminally tagged Cβ2 was kinase inactive and was not used for further experiments (results not shown).

In order to further demonstrate that the cloned sequence expressed a murine Cβ2 (mCβ2) specific sequence, we developed an antiserum by immunizing two rabbits with two mCβ2 specific peptides (Figure 3D). Immunoreactivity of the resulting antiserum (Anti-mCβ2) was tested by immunoblotting using lysates from HEK 293T cells transfected with either mCβ2 or human Cα1 (hCα1) (Figure 3E). This revealed that the size of the protein recognized by the anti-mCβ2 serum was determined to be 47 kDa by comparison with an identical blot probed with the pan C antibody. It should be noted that the anti-mCβ2 serum did not appear to cross-react with expressed hCα1.

## Discussion

The present report demonstrates a lymphoid tissue specific 47 kDa Cβ subunit which together with Cβ1 (40 kDa) is responsible for 66 % of PKA specific enzyme activity in spleen cells. The 47 kDa Cβ subunit was cloned and shown to be an active murine PKA Cβ splice variant. Based on a sequence homology search, and production of a specific antiserum, we conclude that the 47 kDa protein is the murine homologue of human and bovine Cβ2. Furthermore, sequence comparison also led us to conclude that the previously published murine Cβ2 is the murine homologue of human Cβ4. Taken together with previous reports our results demonstrate that the murine Cβ gene encodes Cβ1, Cβ2, Cβ3 and Cβ4.

Expression of Cβ1 in murine spleen was indirectly demonstrated by reduced immunoreactivity of the 40 kDa band and a 27 % reduction in PKA-specific kinase activity in the Cβ1-/- mouse. Cβ1 expression in murine lymphocytes is in conflict with our previous observations in human T lymphocytes [18], which apparently do not express this splice variant. If this is also the case for murine T cells, it implies that other cell types may account for the presence of Cβ1 in our cell extracts, since spleen cells are a mixture of various immune cells, including B and T cells as well as macrophages and monocytes.

Our results demonstrate the existence of a sequence in the murine genome homologous to the human and bovine Cβ2-specific sequence. In addition, we observed a 47 kDa anti-C immunoreactive protein that was absent in Cβ-/- but not in Cβ1-/- mice. Because the cloned and expressed Cβ variant was 47 kDa as demonstrated by immunoreactivity to a pan C antibody and a serum made against the N-terminus of the novel protein, and because the only known 47 kDa PKA C subunit is the human and bovine Cβ2 [17,22], we conclude that the novel C subunit is the murine Cβ2 homologue. The existence of the 47 kDa Cβ2 homologue is in conflict with our previous report, which showed the presence of Cβ2 in several mammalian species, including monkey, dog, man and bovine, but failed to demonstrate the splice variant in rodents [17]. This was concluded based on a Zoo-blot (Southern blot with genomic DNA from various species) analysis using a probe spanning the human Cβ2-specific sequence. Failure to identify a murine Cβ2 may have been due to low homology between Cβ2 of various species, which we also discovered in our data-base searches comparing the Cβ2 specific sequence of human, bovine, mouse and zebra fish (Figure 2C). Interestingly, the Cβ2 specific sequence was fairly well conserved even in the fish genome, indicating the presence of Cβ2 in all vertebrates. A lower of degree of conservation is a feature generally associated with sequences not directly involved in catalysis, which again may suggest a more structural role for the N-terminal part of the protein.

Expression of Cβ2 protein seemed to be mostly restricted to lymphoid tissues, as the 47 kDa band was present in lymph nodes, spleen and thymus, but not detectable in brain, heart, lung, liver, kidney and skeletal muscle. In an earlier report Cβ2 mRNA was detected in bovine brain, heart, skeletal muscle, spleen and liver [14]. Moreover, in human Cβ2 has been detected by Northern blotting in human kidney in addition to the same immune tissues as demonstrated for mouse [17]. The fact that we were unable to detect Cβ2 protein in all tissues described in bovine and in kidney of human may imply that the protein is not present in these tissues or that the level of expression is below the level of detection for the antibodies used here.

Using peptides corresponding to the Cβ2 specific sequence, we made a polyclonal antibody. This antiserum was shown to be Cβ2 specific in immunoblot assays.

Guthrie et al. [16] identified three Cβ splice variants in mouse, with Cβ2 expressed exclusively in the brain, encoding an additional 2 amino acids at the N-terminus by alternative use of exons. It is therefore smaller than human and bovine Cβ2 and is not expressed in the same tissues. In fact, the previously described mouse Cβ2 is identical to the human splice variant designated Cβ4, which is expressed in the brain [17]. Based on this evidence, the previously described murine Cβ2 has now been renamed murine Cβ4. Moreover, based on this and previous reports, we conclude that all mammals contain the isoforms Cβ1, Cβ2, Cβ3 and Cβ4. These facts call for a revision of the present Cβ splice variant nomenclature as given in Table 1.

**Table 1:** Nomenclature and relative sizes of human, bovine and murine Cβ splice variants

Human	Cβ1	Cβ2	Cβ3**	Cβ4**	[23, 17]
Bovine	Cβ1	Cβ2			[13, 14]
Murine (old)*	Cβ1		Cβ3	Cβ2	[15, 16]
Murine (new)*	Cβ1	Cβ2	Cβ3	Cβ4	
Size (kDa)	40	47	38	38	

*The old and new murine nomenclature has been indicated.

**The abc variants of Cβ3 and Cβ4 (Cβ4ab and Cβ 4abc [17] and Cβ3ab, Cβ3abc, Cβ3b and Cβ4b [12]) have not been included in the table.

## Conclusion

We conclude that the murine homologue of human and bovine Cβ2 is expressed in lymphoid cells. The previously published murine Cβ2 [16] is the murine homologue of human Cβ4 [17]. Taken together with previous reports we conclude that the murine Cβ gene encodes Cβ1, Cβ2, Cβ3 and Cβ4.

## Methods

### Mice

Cβ-/- mice on a mixed C57BL/6:129SV/J background [19] were obtained from Professor G. Stanley McKnight (Department of Pharmacology, University of Washington School of Medicine, Seattle, 98195, USA). All use of animals has been approved and registered by the Norwegian Animal Research Authority. Animals were housed in a temperature controlled (22°C) facility with a strict 12 hour light/dark cycle, and allowed the RM1 diet (Special Diet Services Ltd, Witham, Essex, UK) and water *ad libitum *before euthanasia. Heterozygote animals were crossed and offspring genotyped by PCR. The KO allele was detected using a forward primer 5'-ccgcttgggtggagaggctat-3' that hybridizes inside the neomycin cassette that replaced exon 2 by homologous recombination when the KO gene was generated, and a reverse primer 5'-gccctccttgccgcct-3' that hybridizes downstream of exon 2, giving a ~1.4 kb product. The WT allele was detected using a forward primer 5'-cggtgctggcaacagaaggtg-3' that hybridizes upstream of exon 2 and a reverse primer 5'-gaagggaacggaggcgagtga-3' that hybridizes inside exon 2, giving a ~1.2 kb product.

### Preparation of primary human and mouse lymphocytes

Human T cells were purified from buffy coates of healthy blood donors (Ullevaal University Hospital Blood Centre, Oslo, Norway). Written approval was obtained from all donors. First, mononuclear cells were isolated using density gradient centrifugation (Lymphoprep; Nycomed, Oslo, Norway) as described elsewhere [24]. The cells were then enriched for T cells by negative selection using anti-CD14 and anti-CD19 coated magnetic beads (Dynal, Oslo, Norway).

Spleens were dissected from CO_2_-euthanized mice. Single cell suspensions in PBS (Phosphate buffered saline, pH 7.4) were obtained by squeezing tissue through a mesh cup (Sigma), and erythrocytes were removed by lysing in hypotonic buffer (17 mM Tris, 140 mM NH_4_Cl, pH 7.2).

### Homogenization and preparation of protein lysates

Cells were washed in PBS and lysed by sonication on ice in a sucrose-containing lysis buffer (5 mM KH_2_PO_4_, 5 mM K_2_HPO_4_, 250 mM sucrose, 1 mM EDTA, 0.5 % Triton X-100) with 1 mM DTT, 1 mM PMSF and a protease inhibitor cocktail (Sigma). Tissue samples were homogenised on ice in the same buffer using Ultra thurrax (3 × 15 seconds, full speed). Lysates were cleared by centrifugation at 15,000 × g for 15 minutes at 4°C. Protein concentration was determined by the Bradford method (Bio-Rad Laboratories).

### Kinase assay

PKA-specific kinase activity was determined as described previously [25,26]. Briefly, 10 μl lysate (1μg/ml) was added to a 40 μl reaction mix to make a final concentration of 30 μM Kemptide, 200 μM of ATP containing 2 μCi/mmol [γ-^32^P]ATP (Amersham Biosciences), 10 mM MgAc_2_, 20 mM Tris (pH 7.4) in the presence or absence of 5μM cAMP and 30 μM PKI. After incubation for 9 minutes at 30°C, 25 μl of the reaction was spotted onto phosphocellulose paper (P81 Whatman), which was washed four times in 75 mM phosphoric acid and finally in 95% ethanol. Filters were air-dried, immersed in Opti-Fluor (Packard Biosciences) and counted in a liquid scintillation counter.

### SDS-PAGE and immunoblotting

Lysates were separated by SDS-PAGE in 12.5 % gels (Criterion Precast Gels, BioRad) and transferred to PVDF membranes by electroblotting. Membranes were blocked in 5% skimmed milk powder in TBST (Tris-buffered saline containing 0.1 % Tween-20) for 1 h at room temperature, and incubated overnight at 4°C with primary antibodies (pan C antibody (Anti-C, BD Transduction Laboratories) or mCβ2 antiserum (Anti-mCβ2, Sigma)) diluted in TBST. Membranes were washed for 1 h in TBST and further incubated with horseradish peroxidase-conjugated secondary antibodies (MP Biomedicals). Membranes were washed and finally developed using SuperSignal^® ^West Pico Chemiluminescent (Pierce).

### Cloning of mCβ2

A homology search in the murine genome was performed in the National Center for Biotechnology Information (NCBI) BLAST (tblastn) search tool using the human Cβ2 specific peptide sequence. A region similar to the human exon 1–2 sequence was identified and a sequence in the mouse genome was postulated as mouse exon 1–2. Based on this a hypothetical murine Cβ2 cDNA was deduced which contained this sequence and the rest of the known murine Cβ cDNA sequence thatspans the sequence encoded by exon 2 through exon 10. To verify the existence of murine Cβ2 mRNA, total RNA was isolated from a mouse thymus using the RNeasy Mini Kit (Qiagen) and total cDNA was synthesized using the Promega Reverse Transcription System (Promega). Potential full-length mCβ2 cDNAs were amplified with PCR (Pfu Ultra amplification system, Stratagene) using several different oligonucleotide primers corresponding to the 5'-untranslated and 3'-untranslated regions of the predicted cDNA. Three different forward primers (F1: 5'-gctcttagcgtcttggtagg-3', F2: 5'-aaacacttgcagttacttta-3' and F3: 5'-ttctgaaaacacttgcagtt-3') and three different reverse primers (R1: 5'-cccacagaggtcggt-3', R2: 5'-gacctcacgcagttg-3' and R3: 5'-acactgatgacgcagga-3') were combined into nine different primer sets (set 1: F1+R1, set 2: F1+R2, set 3: F1+R3, set 4: F2+R1, set 5: F2+R2, set 6: F2+R3, set 7: F3+R1, set 8: F3+R2 and set 9: F3+R3). Of the primer sets (Sets 1, 3, 4, 6, 7 and 9) that resulted in amplification of a single DNA band of the right size (based on the theoretical lengths of the various PCR products-between 1300 and 2000 bp), Set 1 and 3 were used to amplify mCβ2 cDNA in two parallel PCR reactions. The PCR products were subcloned into the pCR-Blunt-II-TOPO vector (Invitrogen) and sequenced by Medigenomix (Martinsried, Germany).

### Expression of mouse Cβ2

For eukaryotic expression of mCβ2, the mCβ2 cDNA was cloned into the mammalian expression vector pcDNA-DEST40 (Gateway Technology System, Invitrogen), during which primers were made to introduce a Kozak sequence at the '5-end. In addition, two '3-end primers were used to introduce or exclude a stop codon in order to express mCβ2 with or without an N-terminal tag encoded by the expression vector itself (Invitrogen). For primer sequences, see figure 3A. PCR products were subcloned into pENTR/D-TOPO vector and transformed into bacteria. A positive clone was purified by mini-preparation (Jetquick Plasmid Spin Kits, Genomed), digested by restriction enzymes and sequenced. Using the LR-clonase recombination reaction, the insert of the pENTR/D-TOPO vector was recombined into the pcDNA-DEST40 expression vector and transformed into bacteria. Positive clones were selected and purified by mini-preparation (Jetquick Plasmid Spin Kits, Genomed), restriction enzyme digested and sequenced. The new plasmid was designated pcDNA-DEST40mCβ2.

### Transfection

HEK 293T cells were grown in RPMI-1640 medium supplemented with 5 % fetal calf serum. Semi-confluent cells (3.5 × 10^5 ^cells/ml) were transfected with human Cα1 (pcDNA-DEST40hCα1 [18]) or murine Cβ2 (pcDNA-DEST40mCβ2) at a concentration of 2 μg DNA/ml using LipofectAMINE2000 (Invitrogen) as described by the manufacturer. Cells were then incubated for 24 hours before use in experiments.

### Production of mCβ2 antiserum

Antisera production, including peptide synthesis, KLH-conjugation and immunization of rabbits was performed by Sigma-Genosys, UK. Rabbits were co-immunized with two peptides with sequences from the Cβ2 specific N terminal end of murine Cβ2, SRLFHRHSRGTAQE and RAALEDDGLRASEH (see figure 3D). Both peptides contained an additional Cys residue at the C-terminus for thiol-coupling to KLH (keyhole limpet hemocyanin). The conjugate was dissolved in PBS and injected with Freund's adjuvant. For the initial injection, 200 μg of conjugate was injected with Complete Freund's Adjuvant, and for subsequent injections 100 μg of conjugate with Incomplete Freund's Adjuvant was injected. Immunizations were given subcutaneously at a single site every two weeks for a total of 6 times. Blood samples were collected 4 times during this period.

## Abbreviations

WT, +/+, wild type; +/-, heterozygote; KO, -/-, knockout; PKA, cyclic AMP-dependent protein kinase, protein kinase A

## Authors' contributions

**AF **contributed to the design of the experiments and maintained housing, breeding and dissection of the mice except the Cβ1 ablated mice. AF carried out the immunoblot analysis of various tissues isolated from wild type mice as well as lymphoid tissues isolated from Cβ ablated mice. Finally, **AF **performed activity measurements, antibody characterization and wrote and made figures for the manuscript. **HHH **contributed with data base search, cloning of Cβ2, making of the Cβ2 expression vector, heterologous expression of the Cβ2 protein and production of the Cβ2 antiserum, and also contributed to dissection of mice and preparation of cell lysates. **TTH **carried out part of the immunoblot and activity assays and characterization of the Cβ2 antiserum.** PSA **contributed with housing, breeding and dissection of Cβ1 KO mice for the preparation of Cβ1 ablated immune tissue extracts as well as the writing of the manuscript. **SØ **contributed to the design, database search and cloning of mouse Cβ2. **BS **conceived of the study, and participated in its design and coordination and helped to draft the manuscript. All authors read and approved the final manuscript.
